# PEP and TasP Awareness among Italian MSM, PLWHA, and High-Risk Heterosexuals and Demographic, Behavioral, and Social Correlates

**DOI:** 10.1371/journal.pone.0157339

**Published:** 2016-06-13

**Authors:** Gabriele Prati, Bruna Zani, Luca Pietrantoni, Diego Scudiero, Patrizia Perone, Lella Cosmaro, Alessandra Cerioli, Massimo Oldrini

**Affiliations:** 1 Department of Psychology, University of Bologna, Bologna, Italy; 2 Italian League for the Fight against AIDS, Como, Italy; The University of New South Wales, AUSTRALIA

## Abstract

Post-exposure prophylaxis (PEP) has been proposed as a strategy to prevent the acquisition of HIV infection after certain high-risk exposures, and treatment as prevention (TasP) is also being advocated as a means to reduce sexual transmission of HIV. The aim of this study was to investigate the prevalence of PEP and TasP awareness and their demographic, behavioral, and social correlates in Italy. We conducted a cross-sectional survey of 1,028 high-risk heterosexual men and women, 1,874 non-HIV positive MSM (men who have sex with men), and 694 people living with HIV/AIDS (PLWHA). The majority of the participants was aware of PEP and unaware of TasP. MSM were less knowledgeable about PEP and TasP than were PLWHA and more knowledgeable about PEP and TasP than were high-risk heterosexual participants. The variables most consistently associated with PEP and TasP awareness were contact with HIV/AIDS organizations, HIV testing, and HIV stigma. A positive relationship between unprotected sexual intercourse and PEP and TasP awareness was found among high risk heterosexual participants, while this association was not significant among MSM and PLWHA. Because PEP and TasP are currently recommended, effective educational and dissemination strategies are needed to increase the level of knowledge about PEP and TasP.

## Introduction

The use of anti-HIV medications has greatly improved the lives of people living with HIV/AIDS (PLWHA). More recently, much attention has been given to the use of anti-HIV medications as a strategy to prevent or control the spread of HIV infection in a number of ways. Anti-HIV medications are being used for post-exposure prophylaxis or PEP [1, 2] and for their preventive effects when used as treatment, that is, the Treatment as Prevention (TasP) approach [3]. PEP involves taking anti-HIV medications soon after exposure to reduce the chance of becoming HIV positive. TasP denotes HIV prevention methods that use anti-HIV medications in PLWHA to reduce the likelihood of HIV infection.

Without getting into the argument of the effectiveness of PEP and TasP, these approaches raise the question of whether their implementation is effective. Adherence to the medication is required for efficacy for HIV prevention [4]. Indeed the question of how acceptable are anti-HIV medications for the prevention of HIV transmission is essential to introduce and maintain sustainable and effective biomedical prevention methods [5]. The acceptability of a biomedical approach to HIV has been related to the idea that people need accurate information in order to make rational choices about their sexual health [6]. Many theories of behavior change such as the Information–Motivation–Behavioral Skills Model [7] and the Health Belief Model [8, 9] suggest that knowledge plays a pivotal role in decision and behavior. Notwithstanding the importance of knowledge about PEP and TasP, few published studies examine knowledge about PEP and TasP, especially among non-US populations [10]. The available evidence suggests that knowledge about PEP and TasP is limited. In a survey of HIV-uninfected gay/bisexual men in California, 47% reported PEP awareness [11], while another study involving HIV-negative homosexually active men in Sydney, Australia, reported that 54% of participants had heard of PEP [12]. The awareness of PEP availability seems to increase over time: a survey among Australian gay men revealed that the awareness of PEP increased from 23% in 2001 to 64% in 2010 [13]. PEP awareness seems to be less limited than awareness of pre-exposure prophylaxis [14–16]. Pre-exposure prophylaxis is different from PEP and TasP since it involves the use of antiretrovirals by HIV-negative before potential exposure to prevent infection.

Two reviews of the literature examining the acceptability of antiretrovirals for the prevention of HIV revealed a lack of TasP research and that there has been far more acceptability research on pre-exposure prophylaxis than TasP [5, 17]. Qualitative studies revealed limited awareness of TasP [18, 19]. Among PLWHA not currently on treatment in Seattle, Washington, 18% believed that antiretroviral therapy decreases HIV transmission [20].

The first aim of the present study was to explore the knowledge about PEP and TasP in Italy. HIV infection is a public health problem in Italy. A prevalence of about 125,000–130,000 PLWHA (including the undiagnosed HIV population) was estimated in Italy [21]. In 2014, 3,695 newly diagnosed HIV infections have been reported in Italy, resulting in an incidence of 6.1 per 100,000 residents. An increase of sexually acquired cases, especially among MSM (men who have sex with men), has been registered in Italy. Heterosexual transmission is the most commonly reported route of HIV transmission in Italy while MSM are disproportionately affected by HIV/AIDS [22–24]. Because the literature on anti-HIV medications prevention interventions focused largely on MSM [5, 17], to address this research gap, we included high-risk heterosexual men and women. It is recognized that certain groups of people such as MSM have been particularly affected by HIV, while heterosexual people have not. Because PEP rests on the prescription of anti-HIV medications to uninfected persons at high risk of HIV acquisition, we focused on non-HIV positive heterosexual participants who reported at least one unprotected sexual intercourse in the last 12 months with one or more casual partners.

The second aim of the study was to investigate the behavioral, demographic, and social correlates of knowledge about PEP and TasP in Italy. Few studies have investigated the behavioral and demographic correlates of knowledge about PEP but research on the behavioral and demographic correlates of TasP awareness is lacking. Knowledge about PEP was associated with older age, gay/homosexual identity, and having had unprotected anal sex [11]. A previous study suggested that gay community organizations contributed to an increase in knowledge about PEP [13]. In the present study, we tested the hypothesis that the degree of contact with HIV/AIDS organizations is associated with greater knowledge about PEP and TasP. In addition, we expected that HIV stigma is associated with less knowledge about PEP and TasP. Research suggests that stigma poses a significant barrier to effective prophylaxis [25].

Given that knowledge about PEP and TasP is a necessary (although not sufficient) condition for acceptability, the current study sought to elucidate the behavioral, demographic, and social correlates of knowledge about PEP and TasP. Understanding the correlates of knowledge about PEP and TasP is important to provide issue-specific and population specific information.

Another aim of the current study was to investigate the relationship between knowledge about PEP and TasP and frequency of unprotected sexual intercourse with casual partners. Although unprotected sex is reduced among PLWHA on antiretroviral therapy [26], there is evidence that the belief that receiving antiretroviral therapy protects against transmitting HIV is associated with higher prevalence of unprotected sex [27]. Thus, we examined the relationship between the knowledge about PEP and TasP and the frequency of unprotected sexual intercourse with casual partners.

## Materials and Methods

### Participants and Procedures

The research has been conducted according to the principles expressed in the Declaration of Helsinki. The study was approved by the ethics committee of the Department of Psychology of the University of Bologna. Informed consent was obtained via our online system from all participants. After introducing the goals of the study in details the participants were asked to tick into a box if they agreed to continue and participate in the study. Participants' consent was stored in secure server.

To collect data, a national, online survey was conducted in January-October 2014. Participants were eligible if they were at least 18 years old and able to complete the survey in Italian language. Participants were recruited through e-mail lists, social network, and web-based communities. Participants were informed that their participation was voluntary with no incentive other than personal knowledge and contributing to general knowledge. Further details on participant recruitment have been previously published [28, 29].

The sample included 6,781 (72.6%) non-HIV positive heterosexual people, 1,874 (20.0%) non-HIV positive MSM (we considered MSM men who had only male partners or men with both male and female partners in the last 12 months), and 694 PLWHA (7.4%). To focus our analysis on heterosexual persons at high risk of HIV acquisition, we restricted the analysis to non-HIV positive heterosexual respondents who reported at least one unprotected sexual intercourse in the last 12 months with casual partner (*n* = 1,028; high-risk heterosexual men and women).

### Measures

Questions assessing demographics, sexual behavior, sexual orientation, relationships, HIV testing, and HIV status were drawn from existing research [30–32]. Sexual orientation was based on gender of sex partners in the preceding 12 months. Unprotected sexual intercourse was measured by asking participants whether and how often (on a four-point response scale, ranging from never to always) they engaged in unprotected vaginal and anal sex with casual partners during the past 12 months. An ordinal as well as a dichotomous variable (No unprotected intercourse vs. Unprotected intercourse with casual partners) was constructed from the responses. Participants were asked whether they and their steady partner (if applicable) have ever been tested for HIV (answers: ‘yes/no’) and, if so, when and their most recent test result. Participants that did not undergo HIV testing were classified as non-HIV positive (e.g.[33]). To assess knowledge of PEP and TasP, respondents were asked, 1) “Shortly after a potential HIV exposure, does a medical treatment exist that could reduce the chance of becoming HIV-positive?”; and 2) “Do you think that people who take medications for HIV are less likely to give the infection to their sex partners if they have unprotected sex?” To determine participants' level of contact with HIV/AIDS organizations, respondents were asked about how often they had contact with HIV/AIDS organizations on a 5-point scale (1 = *Never*, 5 = *Very often*). We measured stigmatizing attitudes toward PLWHA using a six-item scale [34]. Participants rated each item on a 4-point scale (1 = *Strongly disagree*, 4 = *Strongly agree*). A single-factor structure for this scale of stigmatizing attitudes was supported by the parallel analysis and Velicer's MAP test [35]. The reliability of this scale of stigmatizing attitudes toward PLWHA was acceptable (α = .68; ω = .75). Questionnaire items are reported in the file S1 Questionnaire.

### Missing Data Analysis

Of the 13,873 people who visited the survey link, 12,441 agreed to participate in the study. The final sample was 9,349 since 3,092 (25%) participants were excluded due to incomplete data. We employed the usual procedure for testing missingness for independence (i.e., Little’s MCAR test and separate variance t-tests). Analyses revealed that data were missing at random. As recommended by Graham [36], the multiple imputation technique was used for handling missing data.

### Power Analysis

We used G*Power 3.1.9.2 [37, 38] and PASS 11.0.8 [39] to perform power analysis. Specifically, we calculated the level of power achieved for each of the statistical analyses performed in the present study. Alpha was set to be .05. A small-to-medium effect size was chosen. Sample sizes were the numbers of non-HIV positive heterosexual people, non-HIV positive MSM, and PLWHA. Based on results of power analysis, the sample sizes were sufficiently large since all power levels were greater than .80.

### Statistical Analysis

Before statistical analysis, we inspected manually the database for suspicious or duplicate entries. We used SPSS 23 for analysis. The chosen level of statistical significance was *p* < .05. Univariate differences were assessed with chi square tests, while logistic regression was used to identify the predictors of knowledge about PEP and TasP.

## Results

Demographic and behavioral characteristics are shown in Table 1. Each sample was diverse with respect to gender, age, education, relationship status, HIV testing, and unprotected intercourse with casual partners. Owing to the small sample size, transsexual participants were excluded from the analysis. Among PLWHA, the majority of participants was men. PLWHA were older and less educated than MSM and high-risk heterosexual participants.

**Table 1:** Samples Characteristics.

Variables		PLWHA		High-risk heterosexuals		MSM		Chi-Square Tests
		%	*n*	%	*n*	%	*n*	
Gender								1255.89(2)*
	Male	82.8_a_	414	46.5_b_	478	100_c_	1874	
	Female	16.8_a_	84	53.5_b_	549	—	—	
	Transsexual	0.4	2	0	0	0	0	
Age								318.57(6)*
	18–29	15.0_a_	104	49.0_b_	486	39.7_c_	721	
	30–39	27.1_a_	188	30.0_a_	298	33.3_a_	604	
	40–49	32.1_a_	223	14.0_b_	139	20.1_c_	364	
	50 or older	20.6_a_	143	7.0_b_	69	6.9_b_	126	
Education								80.75(4)*
	Primary or secondary school	47.0_a_	235	26.2_b_	269	31.4_c_	588	
	Bachelor's degree	18.4_a_	92	24.0_b_	246	23.7_b_	443	
	Master's degree	34.6_a_	173	49.8_b_	511	44.9_c_	840	
Italian citizenship		96.6	483	98.1	1006	97.7	1828	2.22(2)
In a relationship		46.3_a,b_	232	43.7_b_	447	51.8_a_	969	17.90(2)*
Sexual orientation								
	Heterosexual	17.2	68	100	1027	0	0	
	Gay	77.3	306	—	—	100	1874	
	Lesbian	0.0	0	—	—	—	—	
	Bisexual	5.56	22	—	—	0	0	
HIV testing in the last year		60.8_a_	422	29.0_b_	298	52.7_c_	988	213.96(2)*
Unprotected intercourse with casual partners		56.1_a_	389	100_b_	1028	70.4_c_	1206	462.96(2)*
Knowledge about PEP		87.6_a_	608	75.5_b_	776	82.3_c_	1543	42.49(2)*
Knowledge about TasP		60.5_a_	420	32.8_b_	337	41.9_c_	785	131.70(2)*

* p >.001. Subscript letters indicate which pairs of columns (for a given row) are significantly different as the results of pairwise comparisons of column proportions (Bonferroni correction was applied).

Using McNemar’s test, we found that high-risk heterosexual participants (χ^2^(1) = 334.81, *p* < .001), MSM (χ^2^(1) = 608.33, *p* < .001), and PLWHA (χ^2^(1) = 137.77, *p* < .001) were more aware of PEP than TasP. The chi-square test showed that PLWHA had a greater knowledge about PEP compared to MSM, who, in turn, were more aware of PEP than high-risk heterosexuals, χ^2^(2) = 42.491, *p* >.001. The same pattern of differences was found in the knowledge about TasP, χ^2^(2) = 131.703, *p* >.001. Since there were demographic differences across the three groups (i.e., PLWHA, High-risk heterosexuals, and MSM), we used the Cochran–Mantel–Haenszel Test which permits tests of association between awareness of PEP or TasP and group membership while controlling for third variables. We included gender, age, and level of education as controlling variables. Results showed that there was still an association between group membership and awareness of PEP [χ^2^_CMH_(1) = 7.746, *p* = .005] or TasP [χ^2^_CMH_(1) = 55.067, *p* > .001] after adjusting for gender, age, and level of education.

In multivariate logistic regression analysis (Table 2), being aware of PEP among PLWHA was associated with higher level of education (OR = 1.99; 1.12–3.55) and more frequent contact with HIV/AIDS organizations (OR = 1.37; CI = 1.07–1.75). Among high-risk heterosexual participants, knowledge of PEP was associated with being younger (OR = 0.56; CI = 0.32–0.98), and having had an HIV test in the last year (OR = 1.75; CI = 1.24–2.47). Among MSM, PEP awareness was higher among those with younger age (OR = 0.47; CI = 0.28–0.78), previous HIV testing (OR = 1.29; CI = 1.00–1.81), higher level of education (OR = 1.37; 1.04–1.81), lower levels of HIV stigma (OR = 0.74; CI = 0.55–0.99), and more frequent contact with HIV/AIDS organizations (OR = 1.32; CI = 1.16–1.50).

**Table 2:** Factors associated with Awareness of PEP.

Predictors		PLWHA		High-risk heterosexuals		MSM	
		AOR	95% CI	AOR	95% CI	AOR	95% CI
Female gender		1.63	(0.50–2.28)	0.95	(0.70–1.28)	—	—
Age							
	18–29						
	30–39	1.67	(0.80–3.49)	0.80	(0.57–1.13)	0.79	(0.58–1.06)
	40–49	1.26	(0.63–2.52)	0.91	(0.58–1.43)	0.69	(0.49–0.96)
	50 or older	1.59	(0.70–3.61)	0.56	(0.32–0.98)	0.51	(0.32–0.84)
Education							
	Primary/secondary school						
	Bachelor's degree	1.13	(0.61–2.11)	1.09	(0.72–1.66)	1.13	(0.81–1.57)
	Master's degree	1.99	(1.12–3.55)	1.11	(0.78–1.59)	1.37	(1.04–1.81)
Italian citizenship		2.17	(0.68–6.94)	1.25	(0.47–3.30)	0.71	(0.29–1.73)
In a relationship		0.89	(0.53–1.49)	1.14	(0.85–1.52)	1.29	(0.99–1.70)
Sexual orientation							
	Heterosexual			—	—	—	—
	Lesbian	1.20	(0.11–12.94)	—	—	—	—
	Gay	1.30	(0.59–2.84)	—	—	—	—
	Bisexual	0.96	(0.30–3.12)	—	—	—	—
HIV testing in the last year		0.66	(0.39–1.12)	1.75	(1.24–2.47)	1.29	(1.00–1.64)
Unprotected intercourse with casual partners		1.52	(0.86–2.69)	—	—	1.02	(0.74–1.40)
HIV stigma		—	—	0.88	(0.65–1.21)	0.74	(0.55–0.99)
Contact with HIV/AIDS organizations		1.37	(1.07–1.75)	1.07	(0.91–1.25)	1.32	(1.16–1.50)

Note. AOR = adjusted odds-ratio; 95% CI = 95% confidence interval.

Table 3 shows the results of multivariate logistic regression analysis of variables associated with TasP awareness. Among high-risk heterosexual participants, awareness of TasP was associated with more frequent contact with HIV/AIDS organizations (OR = 1.17; CI = 1.01–1.35). Among MSM, knowledge of TasP was related to higher levels of education (OR = 1.33; CI = 1.06–1.66), HIV testing in the last year (OR = 1.57; CI = 1.29–1.90), lower levels of HIV stigma (OR = 0.56; CI = 0.42–0.74), and more frequent contact with HIV/AIDS organizations (OR = 1.15; CI = 1.06–1.26). Among PLWHA awareness of TasP was associated with more frequent contact with HIV/AIDS organizations (OR = 1.40; CI = 1.20–1.64), unprotected intercourse with causal partner (OR = 1.58; CI = 1.07–2.33), and high level of education (OR = 1.73; CI = 1.20–2.50). Subsequently, we added HIV serodiscordancy in our logistic regression analysis to investigate whether PLWHA in HIV-serodiscordant couples were more aware of TasP than PLWHA in HIV-seroconcordant couples. We did not find any difference in TasP awareness between PLWHA in HIV-serodiscordant couples and PLWHA in HIV-seroconcordant couples (OR = 0.80; CI = 0.45–1.41).

**Table 3:** Factors associated with Awareness of TasP.

Predictors		PLWHA		High-risk heterosexuals		MSM	
		AOR	95% CI	AOR	95% CI	AOR	95% CI
Female gender		1.34	(0.76–2.37)	0.73	(0.50–1.04)	—	—
Age							
	18–29						
	30–39	1.18	(0.71–1.97)	0.97	(0.70–1.33)	1.17	(0.93–1.47)
	40–49	0.90	(0.54–1.49)	1.41	(0.95–2.10)	1.00	(0.76–1.31)
	50 or older	1.87	(0.48–1.58)	0.78	(0.44–1.40)	0.82	(0.55–1.23)
Education							
	Primary/secondary school						
	Bachelor's degree	1.25	(0.80–1.94)	1.14	(0.77–1.68)	1.11	(0.85–1.45)
	Master's degree	1.73	(1.20–2.50)	1.00	(0.72–1.40)	1.33	(1.06–1.66)
Italian citizenship		1.50	(0.58–3.61)	1.11	(0.42–2.94)	0.71	(0.38–1.32)
In a relationship		1.34	(0.94–1.91)	1.02	(0.78–1.33)	0.85	(0.69–1.05)
Sexual orientation							
	Heterosexual			—	—	—	—
	Lesbian	0.96	(0.18–5.21)	—	—	—	—
	Gay	1.04	(0.56–1.94)	—	—	—	—
	Bisexual	0.96	(0.41–2.22)	—	—	—	—
HIV testing in the last year		0.96	(0.68–1.35)	1.27	(0.95–1.69)	1.57	(1.29–1.90)
Unprotected intercourse with casual partners		1.58	(1.07–2.33)	—	—	1.02	(0.80–1.29)
HIV stigma		—	—	0.88	(0.65–1.19)	0.56	(0.42–0.74)
Contact with HIV/AIDS organizations		1.40	(1.20–1.64)	1.17	(1.01–1.34)	1.15	(1.06–1.26)

Note. AOR = adjusted odds-ratio; 95% CI = 95% confidence interval.

The Mann–Whitney test was used to compare the distributions of the frequency of unprotected sexual intercourse with casual partner between participants who were knowledgeable about PEP and TasP and those who were not. The frequency of unprotected sexual intercourse of PLWHA who were knowledgeable about PEP did not differ significantly from those who were not, *U* = 8394.00, *z* = −0.43, *p* = .966. Also, PLWHA who were knowledgeable about TasP were not significantly more likely to engage in unprotected sexual intercourse than those who were not, *U* = 20426.00, *z* = −0.74, *p* = .462. Among MSM, the frequency of unprotected sexual intercourse was not related to the knowledge of PEP, *U* = 117966.50, *z* = −0.46, *p* = .647, and TasP, *U* = 195831.00, *z* = −0.02, *p* = .987. However, among high-risk heterosexual participants, the frequency of unprotected sexual intercourse was related to the awareness of PEP, *U* = 88881.50, *z* = −2.22, *p* = .028, and TasP, *U* = 106625.00, *z* = −2.22, *p* = .028.

## Discussion

In the present study, we explored knowledge about PEP and TasP and the behavioral, demographic, and social correlates. The current study demonstrated that knowledge of PEP and TasP among Italian MSM, PLWHA, and high-risk heterosexual respondents was limited. These findings are in line with earlier qualitative [18, 19] and quantitative [20] studies reporting limited awareness of TasP. However, TasP awareness among PLWHA was higher than that reported in a previous study [20]. At the time of the study of Dombrowski, Harrington [20], the most up-to-date guidelines suggested that some PLWHA might consider starting antiretroviral therapy early, in part, to prevent HIV transmission. Therefore, the diffusion of new information may explain the difference in awareness between the present study and the study of Dombrowski, Harrington [20] However, that the diffusion of new knowledge takes time is not a sufficient explanation for our findings of modest TasP awareness because this study was conducted in 2014. A strong focus on providing accurate and up-to-date information is needed. An opportunity for the delivery of health-related information is the use of media-based sexual-health interventions [40, 41]. According to Nodin, Carballo-Dieguez [15], social marketing approaches can successfully make use of analogies provided by the targets. For instance, PEP can be associated with the concept of emergency contraception or TasP with the contraceptive pill.

Knowledge about PEP was higher than knowledge about TasP across the three samples. Simply put, the majority of the participants was aware of PEP and unaware of TasP. The only exception was that the majority of PLWHA was aware of TasP. Knowledge of PEP and TasP may reflect general media exposure. In a previous study [15], exposure to media (e.g., televisions series, articles from newspapers or the Internet) played an important role in the case of PEP knowledge.

We found that PLWHA were more knowledgeable about PEP and TasP than MSM. A previous study provided evidence that HIV-negative people are more skeptical about HIV treatments’ efficacy in reducing the likelihood of HIV transmission compared to PLWHA [42]. On the one hand, PLWHA would have more intimate knowledge of HIV treatments including their preventative effects. On the other hand, it is perhaps easier to accept the idea that someone might be HIV-infected but not infectious when attributed to oneself than to others. In this way, unprotected sexual behaviors may respond to the needs for ‘normalized’ social identity and relationships that are threatened by the stigma of HIV infection [19, 43]. In addition, we found that high-risk heterosexual participants were less knowledgeable of PEP and TasP compared to MSM and PLWHA. It is well-known that AIDS as a disease was publicly conceptualized as a ‘gay plague’. This view may have allowed heterosexual people to distance themselves from feeling vulnerable to HIV infection and AIDS as a disease. As a result, heterosexual people may be less informed about HIV/AIDS including the effects of HIV treatments.

The variable most consistently associated with PEP and TasP awareness was contact with HIV/AIDS organizations. Contact with HIV/AIDS organizations may provide opportunities to receive information and discuss issues about HIV/AIDS. Among MSM and high-risk heterosexual participants, HIV testing was associated with higher levels of knowledge. The role of HIV testing may be explained by a greater concern for one’s risk of contracting HIV and familiarity with healthcare services. Among MSM and high-risk heterosexual participants, PEP awareness was associated with younger age. Younger people may be more interested in up-to-date information. In addition, there may be a generational difference in how MSM and high-risk heterosexual people access, receive, and share sexual health information. New digital media (e.g., the Internet, text messaging, and social networking sites) have expanded the options for accessing, receiving, sharing, and disseminating sexual health information, especially for youth [44, 45]. The growth of information and communication technologies in Western countries lead to generational differences in the adoption and use of new media [46].

HIV stigma also played a role in PEP and TasP awareness among MSM, but not among high-risk heterosexual people. We note that gay men have been blamed for the disease since the beginning of the diffusion of AIDS when it was initially considered a gay-related immune deficiency. Therefore, HIV stigma can be perceived more as a threat for social identity among MSM than high-risk heterosexual people. The influence of HIV stigma on misinformation about HIV [47] may be higher among people who perceive their identity as more threatened. Since stigma exists among MSM [48], the negative impact of HIV stigma on PEP and TasP awareness among MSM may be explained by social isolation and a lack of supportive networks.

It is interesting to note that PLWHA in HIV-serodiscordant couples reported similar levels of awareness of TasP compared to PLWHA in HIV-seroconcordant couples. Public health efforts should ensure that PLWHA, especially those living in HIV-serodiscordant couples and less educated, have information about TasP. A previous study revealed that the majority of PLWHA expressed interest in starting HIV treatments specifically to reduce the risk of transmitting HIV to sexual partners [20].

Knowledge of PEP and TasP was not associated with an increase in risk-taking behavior among MSM and PLWHA. Although a previous study provided evidence that knowledge about PEP is associated with having had unprotected anal sex [11], in this study we demonstrated that knowledge of PEP does not automatically translate into risk-taking behavior. Knowledge of PEP and TasP does not necessarily provide a rationalization for engaging unprotected sex [49, 50]. However, among high-risk heterosexual participants, knowledge about PEP and TasP was associated with engaging in unprotected sexual intercourse. Since high-risk heterosexual participants had a poorer knowledge of PEP and TasP compared to MSM and PLWHA, we hypothesize that the association between risk-taking behavior and PEP and TasP awareness among high-risk heterosexual participants may be explained by HIV infectiousness beliefs, that is, the idea that HIV medications reduce the infectiousness of PLWHA [51]. Such beliefs were reported prior to the TasP era and may reflect misconceptions and optimistic biases rather than accurate knowledge among high-risk heterosexual participants. Taken together, the findings concerning the relationship between risk-taking behavior and PEP and TasP awareness across the three groups (i.e., PLWHA, High-risk heterosexuals, and MSM) suggest that knowledge about PEP and TasP is unlikely to explain high-risk sexual behavior [52]. In addition, there is also debate about whether HIV infectiousness beliefs is the cause or the consequence of high-risk sexual behavior as a post-hoc justification or rationalization for unprotected sex [49, 50]. Regardless of this debate, we would like to highlight the importance of providing sexual risk reduction counseling along with education on the preventive effects of anti-HIV medications.

Some research limitations should be acknowledged. First, our study employed a cross-sectional design and causal inferences cannot be made. Second, the role of participant self-selection should be taken into account, meaning that those most interested in the issue of HIV/AIDS were most likely to volunteer. Compared to the Italian adult population [53], the study sample included a higher proportion of men, younger adults, people with higher education levels, and MSM. The participants who responded to adverts to take part in the study may represent individuals that are more informed about HIV/AIDS topics. Nonetheless, the characteristics of MSM and PLWHA participants are similar to samples of Italian MSM and PLWHA [30, 32]. For example, previous studies showed that PLWHA in Italy are predominantly male and a continued ageing of this population has been registered [24, 54]. We note that in the present study differences across the three groups (i.e., PLWHA, High-risk heterosexuals, and MSM) were investigated adjusting for demographic variables.

In conclusion, to our knowledge, this is the first study to report on the prevalence of PEP and TasP awareness and on the demographic, behavioral, and social correlates associated with PEP and TasP awareness among Italian MSM, PLWHA, and high-risk heterosexual participants. Despite guidelines for PEP use and TasP, we found limited knowledge of PEP and TasP. Given low overall awareness, educational initiatives are needed and particular attention should be paid to disseminating information to high-risk heterosexual people. Contact with HIV/AIDS HIV/AIDS organizations appeared to play a significant role in increasing knowledge of PEP and TasP. Also, voluntary HIV counseling and testing services can be useful for disseminating information, given the correlation between HIV testing and PEP use and TasP awareness. The present study has also documented a positive relationship between unprotected sexual intercourse and PEP and TasP awareness among high-risk heterosexual participants, while this association was not significant among MSM and PLWHA.

## Supporting Information

S1 Questionnaire Questionnaire items. (DOCX)
