# Supplementary material for: PEP and TasP Awareness among Italian MSM, PLWHA, and High-Risk Heterosexuals and Demographic, Behavioral, and Social Correlates
Source: PLoS One. 2016 Jun 13;11(6):e0157339. doi: 10.1371/journal.pone.0157339 (PMC4905673; doi:10.1371/journal.pone.0157339)
Supplement: S1 Questionnaire — (DOCX) [file pone.0157339.s001.docx]

Questionnaire items

Please report your socio-demographic information:

Gender

Age

Education

Italian citizenship (yes, no)

Are you in a steady relationship? (yes, no). If yes, have the steady partner ever been tested for HIV (answers: ‘yes/no’) and, if yes, when and their most recent test result.

Did you have an HIV test? (yes in the last year, yes but more than one year ago, never). If yes, please report the results

During the past 12 months, did you have sexual intercourse? If yes, did you have sexual intercourse with (female partner, male partner or both female and male partner)

During the past 12 months, did you have at least one unprotected intercourse with casual partners? (yes, no). If yes, did you engage in…

• unprotected vaginal sex with casual partners during the past 12 months? (yes/no)

• unprotected anal sex with casual partners during the past 12 months? (yes/no)

Shortly after a potential HIV exposure, does a medical treatment exist that could reduce the chance of becoming HIV-positive? (yes, no)

Do you think that people who take medications for HIV are less likely to give the infection to their sex partners if they have unprotected sex? (yes, no)

During the past 12 months, how often did you have contact with HIV/AIDS organizations? (1 = Never, 5 = Very often)

Stigmatizing attitudes. Participants rated each item on a 4-point scale (1 = Strongly disagree, 4 = Strongly agree)

• It is not safe for a person with HIV to look after somebody else’s children

• I feel afraid to be around people with HIV

• People with HIV deserve as much respect as anyone else (reverse)

• I would not drink from a tap if a person with HIV had just drunk from it

• If you have HIV you must have done something wrong to deserve it

• I feel uncomfortable around people with HIV
